# Tolerance to exercise intensity modulates pleasure when exercising in music: The upsides of acoustic energy for High Tolerant individuals

**DOI:** 10.1371/journal.pone.0170383

**Published:** 2017-03-01

**Authors:** Mauraine Carlier, Yvonne Delevoye-Turrell, on behalf of the Fun2move consortium

**Affiliations:** SCALab, UMR CNRS 9193, Department of psychology, University of Lille, Lille, France; University of Zurich, SWITZERLAND

## Abstract

Moderate physical activity can be experienced by some as pleasurable and by others as discouraging. This may be why many people lack sufficient motivation to participate in the recommended 150 minutes of moderately intense exercise per week. In the present study, we assessed how pleasure and enjoyment were modulated differently by one’s tolerance to self-paced physical activity. Sixty-three healthy individuals were allocated to three independent experimental conditions: a resting condition (watching TV), a cycling in silence condition, and a cycling in music condition. The tolerance threshold was assessed using the PRETIE-Questionnaire. Physical activity consisted in cycling during 30 minutes, at an intensity perceived as “somewhat difficult” on the Ratings of Perceived Exertion Scale. While controlling for self-reported physical activity level, results revealed that for the same perception of exertion and a similar level of enjoyment, the High Tolerance group produced more power output than the Low Tolerance group. There was a positive effect of music for High Tolerant individuals only, with music inducing greater power output and more pleasure. There was an effect of music on heart rate frequency in the Low Tolerant individuals without benefits in power output or pleasure. Our results suggest that for Low Tolerant individuals, energizing environments can interfere with the promised (positive) distracting effects of music. Hence, tolerance to physical effort must be taken into account to conceive training sessions that seek to use distracting methods as means to sustain pleasurable exercising over time.

## Introduction

Tolerance to exercise intensity is a trait that influences one’s ability to continue exercising at levels of intensity associated with discomfort and displeasure [1]. As much as there are individuals who like vigorous training sessions and will tolerate high sensory stimulation or pain to some extent (the “Feel strong” profile), many inactive individuals will consider engaging in a physical exercise only at a slow and leisure pace to avoid senses of fatigue, exhaustion or breathlessness. For these reasons, distracting methods (using music and videos) have been developed to decrease the perception of exertion and enhance positive affective states and moods during exercise [2,3,4,5]. Nevertheless, in a typical week, 60% of adults in Europe admit engaging in no physical exercise at all [6]. Worse yet, in the low number of people who choose to initiate a regular program of physical activity, a high rate of dropout has been estimated to be approximately 50% within the first few months [7]. As pleasure seems to be a key feature in motivating exercising, the aim of this study was to assess the effects of a distracting musical environment on the pleasure and the enjoyment experienced during the practice of self-paced cycling activities in sedentary active and inactive individuals.

The distribution of energy expenditure throughout an exercise task is known as pacing and is extremely important as it predetermines the ability to maintain the intensity of a physical exercise across a given period of time. When prescribing physical exercises for health benefits to inactive individuals, care practitioners need to specify the dosage of exercise as a function of many parameters (e.g., frequency (F), intensity (I), duration (time, T) and type (T)–the FITT principle–[8]). However, intensity is thought to be the most important variable to guide cardiovascular training as exceeding the appropriate intensity leads to discomfort, overexertion and injury, possibly leading one to avoid future activity [9,10,11,12]. Conversely, an intensity that is lower than what is recommended may prevent noticeable health and fitness benefits but can also be boring, causing frustration and again possible dropout. This is why the practice of a moderate physical activity at 75% of VO2 max is proposed as a good pace intensity for health benefit physical exercise and low dropout. However, several studies have now reported that when prescribing moderate intensities of practice on the basis of maximal oxygen uptake, some individuals perceive the physical session as pleasant while others perceive it as unpleasant with significantly less positive feelings [13,14,15]. It has been suggested that these differences in affective responses (pleasant vs. unpleasant) may be due to the nature of the metabolic strain associated with the exercise session [16,17,18], with certain participants using predominantly aerobic sources whereas others require substantial anaerobic supplementation [19].

The point of transition between predominantly aerobic energy production and anaerobic energy production (VT) may be a more appropriate point of reference than VO2max when seeking to prescribe pleasurable activities in inactive populations [19–24]. In fact, according to the dual mode theory, the affective responses to exercise intensity is said to be predicted by the VT transition marker [1,25]. More specifically, below the VT, the aerobic metabolism is predominant. This is the case when exercising at low intensities (below heart rate of 85 bpm). The practitioners will experience positive affect because the body is able to maintain homeostasis, i.e., a constant energy stream for exercise, simply by breaking down carbohydrates and fats through the use of aerobic metabolic processes. However, above the VT, the rate of oxygen consumption is too high and the energy production is not fast enough. Hence, the anaerobic metabolism kicks in accompanied by the appearance of fatigue, muscle burning, and pains that may be perceived as negative affective states. In later years, it has thus become evident that a method to determine the VT intensity must be found.

To date, incremental protocols are often used to determine the VT intensity. However, such a protocol requires practicing until volitional exhaustion, which may not always be accepted by athletes and should be avoided when working with inactive populations [26]. In this context, affective states have been suggested as a possible indicator as they are known to be the primary means by which information about critical disruptions of homeostasis and energy regulations enter consciousness [27–32]. The self-evaluation procedure may help participants to detect slight modulations in inner state homeostasis [1], and hence can be used as benchmark to self-regulate effort intensity during exercise [19,33,34]. On this basis, the Borg Scale of Perceived Exertion is often used to match how hard one *feels and experiences the difficulty of a practice* session [35]. Thus, it is a “relative” scale that starts with “no feeling of exertion” (RPE 6), and ends with “very, very hard” (RPE 20). Moderate activities register 11 to 14 on the Borg scale (“fairly light” to “somewhat hard”), while vigorous activities usually rate a 15 or higher (“hard” to “very, very hard”). In the scientific literature, it has been shown that active healthy individuals and athletes are able to use the RPE as a way to produce different physical efforts [36,37,38]. Moreover, during incremental exercises using production procedures, VT appears when the physical activity is produced and perceived as “somewhat difficult” (RPE 13 on the 6–20 Borg Scale) in active males when running [38] and athletes when cycling [37]. Results obtained by Eston and Williams [36] are interesting as they indicate that non-athlete adults can use the Borg scale to self regulate their physical effort as a function of their inner feelings of exertion. Hence, attention towards inner changes in affective states must be encouraged as it may lead inactive participants to detect the risk of loss of homeostasis right before the aerobic-anaerobic VT transition, providing the means to self-pace exercise intensity throughout a training session [19] and maintain the highest intensity possible with tolerable degrees of physical effort and sensorial discomfort.

The corollary theory is a conceptual cognitive framework that can offer a first level of understanding of how inner sensorial information and affective states linked to physiological changes may be used by the central system to detect the risk of loss of homeostasis [39]. Indeed, the corollary theory suggests that the perception of tolerable discomfort and exertion will depend on the match or the mismatch between (1) a predicted sensory information sent directly from the motor to the sensory areas of the brain and (2) the actual sensory information received by the sensory areas of the brain directly from the body [40]. If the actual level of discomfort corresponds to the acceptable predicted one, the intensity of current practice is maintained. However, in the case of a mismatch, the system will modulate the current intensity to regain a match and to insure sustainable activity at ones’ preferred/acceptable pre-set-intensity of practice. Comparing the actual level of discomfort to the acceptable predicted one will lead practitioners to modulate exercise intensity as a function of the signed computed mismatch. It is the case that the Rating of Perceived Exertion Scale (RPE) involves the collective integration of afferent feedback to enable an individual to evaluate how hard or easy an exercise task feels at any given point in time [41]. Hence, self-evaluation scales implicitly lead individuals to orient attention to those prediction errors made upon the predicted changes in inner sensorial states, targeting as much the positive self-awareness of being, than the negative senses of fatigue, muscle strain and heart pounding.

When considering individuals who possess similar physiological capacities, studies have reported that participants will select a wide range of exercise intensities when tested in self-paced protocols [33, 42]. For example, Lind, Joens-Matre, and Ekkekakis [33] observed that middle-aged, healthy but sedentary women selected intensities varying from as low as 60% to as high as 160% of maximal oxygen uptake as identified from a previous graded-volitional exhaustion treadmill test. Variability in these self-paced intensities of practice may be due to heritable variations in pain sensitivity and tolerance [43] but also in the predicted levels of acceptable sensory stimulations [44,45]. Thus, some individuals will set higher thresholds of practice intensities because they predict liking vigorous training sessions and hence, predict that they will tolerate high sensory stimulations associated to pain and heart rate pounding, while others will prefer intuitively engaging in a physical exercise at a low intensity to minimize discomfort, body temperature and heart rate increases.

The corollary theory postulates also that the environment will influence body states and as such, will influence the actual sensory information that the brain truly receives [39]. Thus, modified environments, e.g., presence or absence of music, will possibly modulate the mismatch error between predicted and actual perceived sensory afferences. Music has an entrainment effect that has been well documented in the past ten years. The Embodied Music Cognition model [46], for example, describes how music and specifically, the groove effect can increase body rhythmicity [47] and modulate body posture [48]. Thus, individuals who tolerate high sensory stimulations may experience as pleasant a physical practice in a musical environment while for others, practicing in such condition could be more challenging than practicing in silence, because of being entrained to cycle at a greater intensity than that expected.

In the present study, the working hypothesis is that for similar self-reported physical activity levels, Low Tolerant individuals will have weaker resistance to sensorial feelings of discomfort than High Tolerant individuals and thus, will self-pace the physical session at lower intensities. We also hypothesised that the distracting effects of music will be similar in Low and High Tolerant individuals. Through the entrainment effects of music, all practitioners should increase exercise intensity. However, unlike the High Tolerant individuals, the music will lead Low Tolerant individuals to exercise outside of their comfort zone and report unpleasant affective states even if practicing within an enjoyable environment. In addition, due to the distracting effects of music, Low Tolerant practitioners may miss the cues indicating the loss of homeostasis benchmark that is critical to offer the feel-good experience of physical exercise.

## Materials and methods

### 2.1 Participants

A total of 68 subjects volunteered to take part in the study but only 63 subjects came (*M*_*age*_ = 22,85 ± 4,78 years, *M*_*BMI*_ = 24,89 ± 5,76 kg.m-^2^). Forty-one women and 22 men were allocated to three independent experimental conditions. All subjects obtained a medical certificate from their medical physicians. Before the beginning of the study, each volunteer read an information letter and completed a written consent form. The study was approved by the Ethics Committee for behaviour human studies of the University of Lille. Participants were asked not to participate in any physical training 48 hours before their inclusion.

### 2.2 Procedure and questionnaires

**Fig 1:**
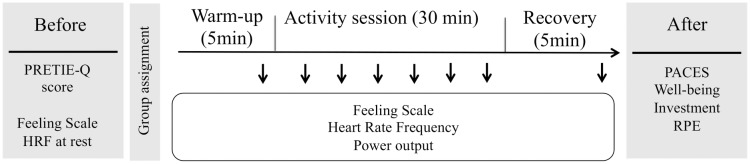
Testing diagram to specify the experimental design that was used in the present study. The different measures taken before, during and after are specified.

The level of self-reported physical activity was assessed using the International Physical Activity Questionnaire (IPAQ–[49]). After reading and completing the consent form, a heart rate monitor (Polar Team^2^—Polar Electro Oy, Kempele, Finlande) was fitted to the participants’ chest and heart rate was recorded during 15 minutes at rest, and throughout the experimental session. After measuring mean heart rate at rest, participants were asked to complete a series of questionnaires in order to obtain socio-demographic data as well as an overall description of tolerance and pleasure. A testing diagram is presented in Fig 1 to illustrate the experimental design.

#### 2.2.1 Self-reported physical activity level assessment [49]

The IPAQ (International Physical Activity Questionnaire) was used to assess the amount of physical activity practiced by our participants. The quantity of physical activity practiced by each participant was calculated by considering the intensity of the reported physical activity sessions (expressed in METs—Metabolic Equivalent of Task—for 3 different categories: low vs. moderate vs. vigorous) as a function of duration (time in minutes) and of the number of days declared per week (METs-minute/week). The feature of this questionnaire is to consider the overall self-reported physical activity level (including daily activities) and not only the physical activity practiced during leisure sports time. Four different measures were obtained: (1) the Total Physical Activity score, which contains the total amount of physical activity practiced (e.g. METs-minute/week__Low_, METs-minute/week__Moderate_ and METs-minute/week__Vigorous_), (2) the Total Low Physical Activity score, (3) the Total Moderate Physical Activity score and (4) the Total Vigorous Physical Activity score.

#### 2.2.2 Preference for and tolerance to the intensity of exercise questionnaire *(French version* [1]*)*

The PRETIE-Q was used to assess the variables of preference for and tolerance to exercise intensity. The eight-item Tolerance scale contained four items that targeted high exercise tolerance (e.g. “I always push through muscle soreness and fatigue when working out”) and four that targeted low exercise tolerance (e.g. “During exercise, if my muscles begin to burn excessively or if I find myself breathing very hard, it is time for me to ease off”). Each item was composed of a 5-point response scale (1 = “I totally disagree”; 2 = “I disagree”; 3 = “Neither agree or disagree”; 4 = “I agree”; 5 = “I strongly agree”). A high score of tolerance to exercise corresponds to a high capacity to pursue the physical activity although it becomes uncomfortable or unpleasant [1]. To obtain two contrasting groups, a median tolerance score was calculated across all experimental groups. Participants with scores lower than the median (27) were considered as Low Tolerant; individuals with scores greater than the median were classified as High Tolerant. After assessing tolerance level, participants were assigned to three independent experimental conditions: a resting TV condition, a cycling in silence condition or a cycling in music condition. In the resting TV condition, there were 10 Low Tolerant individuals and 5 High Tolerant individuals. Both in the cycling in silence and the cycling in music conditions, there were 12 High Tolerant individuals and 12 Low Tolerant individuals.

#### 2.2.3 Feeling Scale [50]

was used to assess the changes in affective states (pleasure / displeasure). This scale consists in a 11-point, single-item, bipolar scale, used for the assessment of affective responses during exercise. The scale ranges from −5 to +5. Anchors are provided at zero, and at all odd integers (+5 = very good; +3 = good; +1 = fairly good; 0 = neutral; −1 = fairly bad; −3 = bad; −5 = very bad).

#### 2.2.4 Physical activity enjoyment scale [51]

The PACES was administered 10 minutes after the end of the experimental session. The questionnaire is composed of 10 items following a 7-point bipolar scale (e.g., *I dislike it—I like it*). Higher scores reflect greater levels of enjoyment. The PACES items were adapted in order to also be used in the resting TV condition.

#### 2.2.5 Investment assessment

was achieved using a 9-point, bipolar scale that was created to get a feel of how much practitioners felt involved in the task. Participants were asked 10 minutes after the end of the session whether they felt to have been invested in the activity, cognitively and physically. The scale ranged from -4 to +4. Anchors are provided at zero and at all even integers (-4 = not at all; -2 = hardly; 0 = neutral; +2 = a lot; +4 = to a large degree).

#### 2.2.6 Well-being assessment

The 5-point SAM Scale was used to assess the state of well-being 10 minutes after the end of the experimental session. Participants were simply asked whether they felt that the session had done them good (e.g., *Yes-I totally agree—No-I totally do not agree*).

#### 2.2.7 Perception of exertion assessment [35]

The Ratings of Perceived Exertion was used 10 minutes after the end of the experimental session to verify that participants considered having done a “somewhat difficult” physical activity throughout the session (RPE 13 on the 6–20 Borg Scale).

### 2.3 Experimental conditions

Following the scores obtained in the PRETIE-Q questionnaire, participants were evenly assigned to three independent experimental conditions: a resting TV condition, a cycling in silence or a cycling in music condition.

In the resting TV condition, participants watched a 40-minute TV documentary relative to newly discovered civilization in Egypt [52]. This experimental condition was chosen in order to control whether the tolerance level may have an impact on the cardiac frequency and affective states and their dynamics throughout a 40-minute period of watching a screen. In the two cycling conditions, participants performed 40 minutes of physical activity. Three phases were proposed: a warm-up (5 minutes), a physical activity (30 minutes), and a recovery period (5 minutes)–following criterion described in ACSM [8].

For all three experimental conditions, changes in the affective states were assessed using the Feeling Scale (FS). In the cycling conditions, the FS was administered before warm up, after warm-up, and every 5 minutes during the practice phase. Thus, nine periods were identified: one before warm-up (0'), one after warm-up (5’), six during the test (10', 15', 20', 25', 30’, 35’), and one after recovery (40'). For all participants, debrief was systematically conducted after the experimental session to explain the aims and the construct of the study.

In the two physical activity conditions, the participants pedalled an electronically ergo-cyclometer (EXC NewBike 700SP, Technogym, Italy). A picture of a path through a forest was projected on a screen (195 cm x 280 cm) placed 250 cm in front of the participants. Pleasant light was also proposed to optimize the pleasant experience of the physical activity [53]. During the warm-up phase, participants were asked to pedal at a speed that would allow them to warm-up and to become familiar with the ergo-cyclometer. During the physical activity (30 min), participants were required to pedal at a speed that they felt as "somewhat difficult" on the Borg RPE scale (RPE 13 on the 6–20 Borg Scale). The power-output (PO) and the heart rate frequency (HRF) produced during the three phases were measured every minute. Nevertheless, for the statistical analyses, PO and HRF were resampled at one sample every 5 minutes.

To create a more pleasurable environment, music was chosen by the participants amongst four "Sport" playlists that were downloaded from the music streaming platform *Spotify*: “motivation for sports”, “power workout”, “cardio” or “sports”. For the warm-up and the recovery phases, music was downloaded from the playlist “Keep calm and stretch it”. A stereo system was used and set at a confortable sonic level for each individual (Yamaha hs 8). The properties of these audio playlists are available as supplementary materials (S1 Table).

### 2.4 Statistical analyses

ANCOVAs were conducted to reveal main effects of Experimental Condition (Resting, Cycling in silence versus Cycling in music) and Tolerance Level (High versus Low Tolerant) while controlling for the Total Physical Activity practiced: (1) on general demographics; (2) on the mean scores of PACES and Well-being; (3) on the mean scores of the perception of exertion scale and the sense of investment felt in the two physical activity conditions. In the resting TV condition, we conducted a mixed model ANCOVA with Tolerance Level (High versus Low Tolerant) as independent factor and Assessment Time (8) as repeated factor, while controlling for the Total Physical Activity practiced. In the two cycling conditions, mixed Model ANCOVAs were conducted to determine effects of Experimental Condition (Cycling in silence versus Cycling in music), Tolerance Level (2) and Assessment Time (8) on Feeling Scale (FS), Heart Rate Frequency (HRF) and Power Output (PO), while controlling for the Total Physical Activity practiced. For the FS and HRF variables, baseline values were systematically included within the statistical model as covariates. Throughout these analyses, partial eta squares (η2p) were calculated to report the effect sizes. Bonferroni-adjusted pairwise comparisons were used when required for the post-hoc analyses.

## Results

### 3.1 Group demographics

The participants that were allocated to the three independent experimental Conditions did not differ in Age (F(2,59) = 1.473, p = 0.237), Body Mass Index (F(2,59) = 0.465, p = 0.630), Educational Level (F(2,59) = 1.467, p = 0.239). No group effects were observed for the IPAQ scores obtained on the dimensions Total Physical Activity (F(2,61) = 0.637, p = 0.532), Total Low (F(2,61) = 0.686, p = 0.507), Total Moderate (F(2,61) = 0.594, p = 0.555) and Total Vigorous Physical Activity (F(2,61) = 0.867, p = 0.425). There was an absence of Tolerance effects on all demographics. Further descriptive results are presented as supplementary material in S2 and S3 Tables.

### 3.2 Results for the individuals watching TV

#### Baseline measures

Main effects of Tolerance Level were found neither on the FS (F(1,12) = 1.532, p = 0.240) nor on the HRF (F(1,8) = 1.235, p = 0.299). Hence, the Low and the High Tolerance groups were similar concerning their affective states and their heart rate frequency before the start of the TV session.

#### Session measures

Tolerance Level main effects were revealed neither on the FS (F(1,11) = 1.133, p = 0.309) nor on the HRF (F(1,7) = 1.949, p = 0.205). No Assessment Time effects were revealed on the FS (F(7,77) = 1.329, p = 0.248). An Assessment Time effect was found on the HRF (F(7,49) = 2.286, p = 0.043, η^2^_p_ = 0.25): HRF was higher at the start (*M*_*HR*_ = 86.4 bpm; *SD* = 13.9 bpm) than at the end of the session *(M*_*HR*_ = 76.3 bpm; *SD* = 9.9 bpm). The interaction Assessment Time x Tolerance Level reached significance neither on the FS (F(7,77) = 0.235, p = 0.975) nor on the HRF (F(7,49) = 1.250, p = 0.295).

### 3.3 Results for the individuals cycling in silence and in music

#### Baseline measures

The main effect of Condition was significant neither for the FS (F(1,43) = 2.948, p = 0.093) nor for the HRF (F(1,31) = 0.157, p = 0.694). No Tolerance Level main effects reached significance for the FS (F(1,43) = 0.249, p = 0.620) and for the HRF (F(1,31) = 0.006, p = 0.937). No Condition x Tolerance Level interactions were found for the FS (F(1,43) = 0.004, p = 0.947) and for the HRF (F(1,31) = 4.006, p = 0.054). Hence, the Low and the High Tolerance groups were similar concerning their affective states and their heart rate frequencies before the start of practice both in the cycling in silence and in the cycling in music conditions.

#### Warm-up measures

The measures taken on the FS during this period revealed an absence of main effects both for Condition and Tolerance Level. A similar absence of effects was revealed for the HRF. Statistical results are presented in Table 1. For the PO, an Assessment Time effect was revealed (F(4,172) = 4.599, p = 0.001, η^2^_p_ = 0.10): the PO was greater at the end (*M*_*Power-output*_ = 52.46 watts, *SD* = 23.9 watts) than at the start of the warm-up period (*M*_*Power-output*_ = 47.07 watts, *SD* = 21.1 watts). No other effects were significant.

**Table 1:** Overall statistical results for the main effects and the interactions on Feeling Scale, Heart Rate Frequency and Power Output as a function of Assessment time, Experimental Condition and Tolerance group.

	Warm up Period				Physical activity at RPE 13				Recovery Period			
	*F*	*df*	*p*	η^2^_p_	*F*	*df*	*p*	η^2^_p_	*F*	*df*	*p*	η^2^_p_
**Main effects**												
*Feeling Scale*												
Experimental Condition effect	0.141	1,42	0.709	0.00	0.718	1,42	0.402	0.02	2.17	1,42	0.148	0.05
Tolerance effect	0.201	1,42	0.656	0.00	0.014	1,42	0.908	0.00	1.069	1,42	0.307	0.02
Assessment Time					0.257	5,210	0.935	0.00				
*Heart Rate Frequency*												
Experimental Condition effect	0.567	1,30	0.457	0.02	1.414	1,30	0.244	0.05	5.808	1,30	**0.022**	0.16
Tolerance effect	1.816	1,30	0.187	0.06	0.734	1,30	0.398	0.02	0.006	1,30	0.938	0.00
Assessment Time	0.388	4,120	0.817	0.01	0.907	5,150	0.478	0.03	0.373	4,120	0.827	0.01
*Power Output*												
Experimental Condition effect	3.469	1,43	0.069	0.07	2.536	1,43	0.118	0.06	5.971	1,43	**0.018**	0.12
Tolerance effect	0.477	1,43	0.493	0.01	5.107	1,43	**0.028**	0.11	4.821	1,43	**0.033**	0.10
Assessment Time	4.599	4,172	**0.001**	0.10	3.634	5,215	**0.004**	0.08	7.368	4,172	**<.001**	0.15
**Interactions effects**												
*Feeling Scale*												
Experimental Condition*Tolerance	0.939	1,42	0.337	0.02	4.161	1,42	**0.047**	0.09	5.304	1,42	**0.026**	0.11
Assessment Time*Experimental Condition					0.631	5,210	0.675	0.01				
Assessment Time*Tolerance					1.946	5,210	0.088	0.04				
Assessment Time*Experimental Condition*Tolerance					0.139	5,210	0.983	0.00				
*Heart Rate Frequency*												
Experimental Condition*Tolerance	0.683	1,30	0.415	0.02	0.15	1,30	0.701	0.00	0.298	1,30	0.589	0.00
Assessment Time*Experimental Condition	1.342	4,120	0.258	0.04	3.786	5,150	**0.003**	0.11	0.943	4,120	0.442	0.03
Assessment Time*Tolerance	1.146	4,120	0.338	0.04	0.697	5,150	0.626	0.02	1.618	4,120	0.174	0.05
Assessment Time*Experimental Condition*Tolerance	0.621	4,120	0.648	0.02	1.287	5,150	0.273	0.04	1.723	4,120	0.149	0.05
*Power Output*												
Experimental Condition*Tolerance	0.746	1,43	0.392	0.02	1.094	1,43	0.301	0.02	0.587	1,43	0.447	0.01
Assessment Time*Experimental Condition	0.914	4,172	0.457	0.02	0.462	5,215	0.804	0.01	2.992	4,172	**0.020**	0.07
Assessment Time*Tolerance	1.114	4,172	0.352	0.03	5.833	5,215	**<.001**	0.12	0.146	4,172	0.964	0.00
Assessment Time*Experimental Condition*Tolerance	0.613	4,172	0.654	0.01	3.599	5,215	**0.004**	0.08	1.288	4,172	0.276	0.03

#### Practice measures

**Fig 2:**
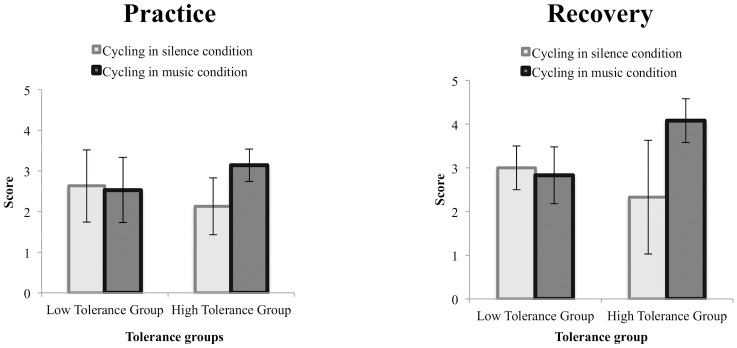
Scores obtained on the Feeling Scale (FS) in the High and Low Tolerance groups during the practice phase (*left*) and the recovery phase (*right*) as a function of the experimental condition (cycling in silence and cycling in music).

Participants were required to cycle at moderate level (RPE 13) for a total period of 30 minutes. When considering the mean FS scores, results revealed an absence of main effects for Condition, Tolerance Level and Assessment Time (Table 1). However, the interaction Condition x Tolerance Level was significant (F(1,42) = 4.161, p = 0.047, η^2^_p_ = 0.09). The Low Tolerance groups revealed similar scores on the FS whether cycling in music (*M*_*Feeling Scale*_ = 2.53, *SD* = 1.58) or in silence (*M*_*Feeling Scale*_ = 2.63, *SD* = 0.89) (p = 0.846). On the other hand, in the High Tolerant groups, greater FS scores were observed in the cycling in music condition (*M*_*Feeling Scale*_ = 3.14, *SD* = 0.89) than in the cycling in silence condition (*M*_*Feeling Scale*_ = 2.13, *SD* = 1.37) (p = 0.051) (Fig 2-*left*).

**Fig 3:**
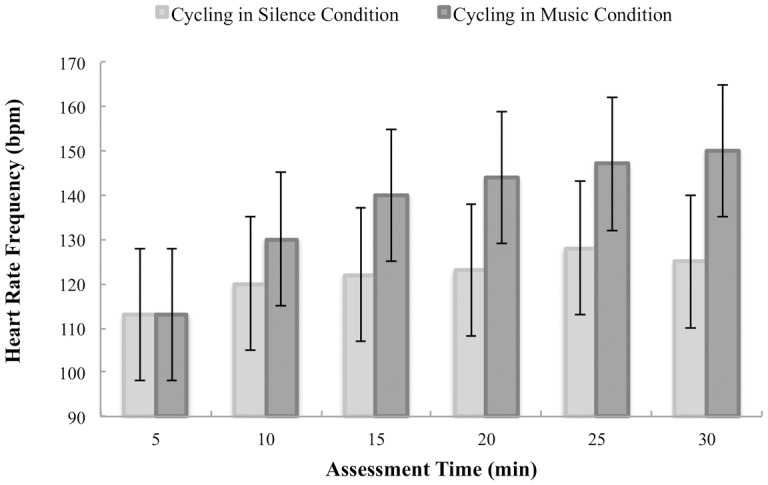
Variations in heart rate frequency as a function of Assessment Time for the cycling in silence condition (*grey*) and for the cycling in music condition (*black*). Error bars illustrate the confidence intervals 95% around the mean.

When considering the HRF, results revealed an absence of main effects of Condition, Tolerance Level and Assessment Time. However, the interaction Assessment Time x Condition was highly significant (F(5,150) = 3.786, p = 0.003, η^2^_p_ = 0.11). For both the Low and High Tolerance groups, HRF increased faster for individuals in the cycling in music condition compared to participants in the cycling in silence condition (Fig 3). In the cycling in music condition, pairwise comparisons revealed greater differences when comparing HRF at different times during the session than in the cycling in silence condition (S4 Table for more details).

For the PO, there were no main effects of Condition but the main effect of Tolerance Level was significant (F(1,43) = 5.107, p = 0.028, η^2^_p_ = 0.11): the High Tolerance group produced more power output (*M*_*Power-output*_ = 88.81 watts, *SD* = 32.42 watts) than the Low Tolerance group (*M*_*Power-output*_ = 70.43 watts, *SD* = 23.13 watts). An Assessment Time effect was also observed (F(5,215) = 3.634, p = 0.004, η^2^_p_ = 0.08): the PO was greater at the end (*M*_*Power-output*_ = 81.98 watts, *SD* = 39.5 watts) than at the start of the practice phase (*M*_*Power-output*_ = 76.33 watts, *SD* = 28.38 watts). The interaction effect between Assessment Time and Tolerance Level was also significant (F(5,215) = 5.833, p < 0.001, η^2^_p_ = 0.12). Pairwise comparisons confirmed significant differences between the High Tolerance group and the Low Tolerance group at 10’ (Delta_means_ = 17.42, SE = 7.8, p = 0.031), at 15’ (Delta_means_ = 16.75, SE = 8.36, p = 0.051), at 20’ (Delta_means_ = 19.29, SE = 8.46, p = 0.028) and at 30’ (Delta_means_ = 32.13, SE = 10.11, p = 0.003) of the physical session, with the High Tolerance group producing always more PO than the Low Tolerance group. Results also revealed in the High Tolerance group significant differences between PO at 30’ and at 5’ of practice (Delta_means_ = 14.38, SE = 4.28, p = 0.019), with more physical output produced at 30’.

**Fig 4:**
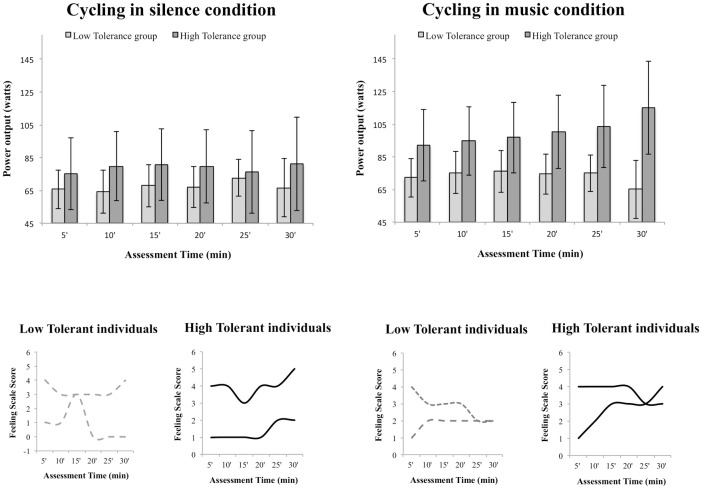
Variations in power output and Feeling Scale. Variations in power output (*Top*) as a function of Assessment Time for the Low Tolerance groups (*grey*) and the High Tolerance groups (*black*) in the cycling in silence condition (*left*) and the cycling in music condition (*right*). Error bars illustrate the confidence intervals 95% around the mean. Scores obtained on the Feeling Scale throughout the 30’ exercise session are presented (*Bottom*) for 8 typical individuals in order to illustrate the non-linearity of the modulations in affective states, whether cycling in silence or in music.

Finally, for the PO, the triple interaction Assessment Time x Condition x Tolerance Level was significant (F(5,215) = 3.599, p = 0.004, η^2^_p_ = 0.08). Results are presented in Fig 4. In the High Tolerance group cycling in music, the PO observed at 30’ of practice differed from that observed at 5’ (Delta_means_ = 22.92, SE = 6.05, p = 0.007), at 10’ (Delta_means_ = 20.08, SE = 5.49, p = 0.010) and at 15’ (Delta_means_ = 18.08, SE = 5.47, p = 0.029), with always more power output produced at 30’ compared to the other assessment times. Post-hoc analyses also confirmed differences between the High Tolerance group and the Low Tolerance group in the cycling in music condition at 20’ (Delta_means_ = 25.75, SE = 11.97, p = 0.037), at 25’ (Delta_means_ = 28.42, SE = 13.34, p = 0.039) and at 30’ (Delta_means_ = 49.83, SE = 14.3, p = 0.012). For the High Tolerance groups only, post-hoc analyses across conditions revealed differences between cycling in music and cycling in silence condition at 25’ (Delta_means_ = 27.33, SE = 13.34, p = 0.047) and at 30’ (Delta_means_ = 33.92, SE = 14.29, p = 0.022), with always more PO produced when cycling in music compared to cycling in silence (S5 Table for more details).

#### Recovery measures

When considering the FS, neither the main effect of Condition nor the main effect of Tolerance Level was significant (Table 1). The interaction Condition x Tolerance Level reached significance (F(1,42) = 5.304, p = 0.026, η^2^_p_ = 0.11; Fig 2*-right*). The Low Tolerance groups were characterized by similar scores on the FS whether cycling in music (*M*_*Feeling Scale*_ = 2.83, *SD* = 1.27) or in silence (*M*_*Feeling Scale*_ = 3, *SD* = 1) (p = 1.00). On the other hand, the High Tolerant individuals in the cycling in music condition presented greater FS scores (*M*_*Feeling Scale*_ = 4.08, *SD* = 1) than the High Tolerant individuals in the cycling in silence condition (*M*_*Feeling Scale*_ = 2.33, *SD* = 2.06) (p = 0.008).

When considering the HRF, no main effects of Condition, Tolerance Level and Assessment Time reached significance. No interactions were found (Table 1).

When considering the PO, results revealed a main effect of Assessment Time (F(4,172) = 7.368, p < 0.001, η^2^_p_ = 0.15), indicating that for all experimental conditions the PO was lower at the end (*M*_*Power-output*_ = 35.67 watts, *SD* = 20.27 watts) than at the start of the recovery phase (*M*_*Power-output*_ = 41.41 watts, *SD* = 27.98 watts).

### 3.4 Perception of physical effort and investment after practice

When subjects were asked to score their levels of effort, there was an absence of differences between the High and Low Tolerance groups (F(1,35) = 0.159, p = 0.693). In addition, the effect of Condition (F(1,35) = 0.013, p = 0.911) and the interaction Condition x Tolerance Level did not reach significance (F(1,35) = 0.150, p = 0.701). When asked to score their feelings of investment, there was also an absence of mean differences between the High and Low Tolerance groups (F(1,43) = 0.067, p = 0.797). Individuals felt to have been invested in the same way both when cycling in silence and when cycling in music (F(1,43) = 1.026, p = 0.317), whether considered as High or Low tolerant individuals (F(1,43) = 2.364, p = 0.131).

### 3.5 Enjoyment and well-being

**Fig 5:**
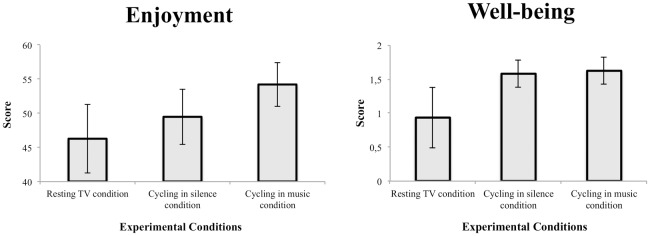
Scores of enjoyment (*left*) and well-being (*right*) are presented as a function of the experimental condition: resting TV condition, cycling in silence and cycling in music conditions. Mean results for the Low and High Tolerance groups are presented to illustrate the absence of group effect.

The PACES enjoyment results revealed a significant effect of Condition (F(2,56) = 3.659; p = 0.032, η^2^_p_ = 0.12), with the cycling in music condition having reported greater levels of enjoyment than the resting TV condition (Fig 5-*left*). Neither the main effect of Tolerance Level (F(1,56) = 1.104, p = 0.298) nor the interaction Condition x Tolerance Level (F(2,56) = 0.482, p = 0.620) were significant. The well-being results revealed a main effect of Condition (F(2,56) = 7.207, p = 0.002, η^2^_p_ = 0.20) with the cycling in music condition and the cycling in silence condition reporting greater levels of well-being than the resting TV condition (Fig 5*-right*). Neither the main effect of Tolerance Level (F(1,56) = 0.972, p = 0.328) nor the interaction Condition x Tolerance Level (F(2,56) = 1.539, p = 0.223) reached significance.

## Discussion

In the present study, we proposed a self-paced cycling activity to both active and inactive sedentary individuals. Using the Ratings of Perceived Exertion Scale (RPE), we allowed participants to set exercise intensity to their own perception of exertion and contrasted power output and affective states when participants were cycling in silence and cycling in music. The main results of our work showed that both High and Low Tolerant participants reported to have (1) exercised at a moderate intensity felt as « somewhat difficult », i.e., RPE 13 on the 6–20 Borg Scale and (2) invested the task at a similar degree of implication. Thanks to the use of a control condition during which participants cycled in silence, we confirmed that the RPE scale can be used as a subjective guide to gauge effort both by High and Low Tolerant individuals as they self-paced the cycling activity to produce similar levels of power output, at the beginning of the session. These results confirm previous studies showing that non-athletes men and women are able to self-regulate physical effort on the basis of a score selected on the RPE scale [37,26]. However, it is during the course of the physical practice that differences between the two groups of participants emerged.

### 4.1 Predicting acceptable intensity of practice

Our results showed that High Tolerant participants were characterised by greater power output compared to the Low Tolerant individuals throughout the 30-min exercise session, confirming again previous studies indicating that ones’ tolerance to exercise is correlated to physical production with more power output produced by those individuals having higher tolerance scores [54]. Our work extends previous studies by confirming tolerance effects on power output while controlling statistically for self-reported physical activity level (as evaluated using the IPAQ). Indeed, in the present study, we characterised the participants by the total quantity of physical activity performed during a typical week across three categories of exercise intensities, which included for example the time spent per week doing house work, gardening, and walking to work in addition to the information classically noted on the number of hours per week spent doing leisure sports. By doing so, we revealed that it may be the individuals’ tolerance level (and not fitness or physical activity level) that predicts the choice in exercise intensity.

**Fig 6:**
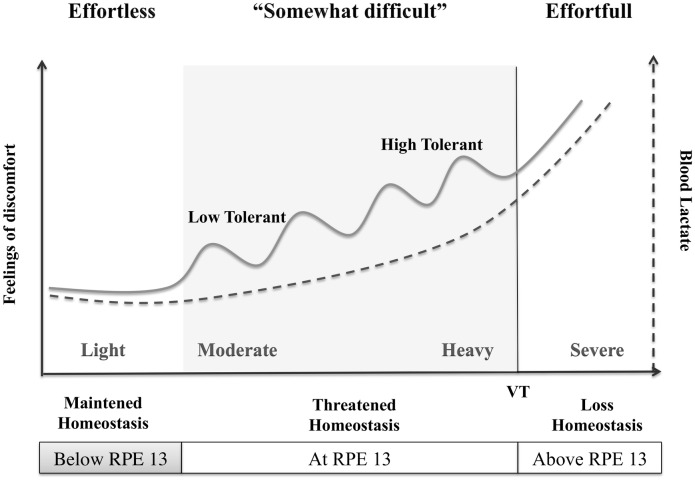
A psycho-physiological framework of tolerance and affective states. This conceptual framework illustrates the possible functional association between biological (lactate concentration—not measured here), physiological (Rating of Perceived Exertion—RPE scale) and psychological factors (Feelings of discomfort; Tolerance to effort). Setting the exercise intensity to “moderately difficult” (RPE13), feelings of discomfort will augment with increasing concentrations of lactate in the blood. As a function of one’s tolerance to inner states of sensorial distress, individuals will target different pre-defined levels of acceptable discomfort, which may be used to detect the upcoming loss of homeostasis. Understanding the psychological factors that modulate one’s ability to set and predict correctly one’s tolerable level of sensorial discomfort may help us gain a better understanding of why some like it slow and easy, and others like it vigorous.

Contrary to the Low Tolerant individuals, High Tolerant adults demonstrated the overall tendency to increase exercise intensity progressively during the course of the exercise session. Setting the exercise intensity to “moderately difficult” but on the low end of a continuum, these participants may have detected a mismatch, i.e. a level of discomfort smaller than that expected when considering their predicted tolerance threshold (see Fig 6). Hence, intensity of practice was augmented progressively to reduce the discrepancy between true and predicted inner states of discomfort. This process may have led the High Tolerant participants in the present study to reach the high end of the moderate continuum by the end of the session, while maintaining good levels of positive affective states. The Low Tolerant group on the other hand may have found a direct match between predicted and true inner states of discomfort leading them to maintain a constant intensity of practice throughout the cycling sessions, remaining at the low end of the moderate intensity continuum.

### 4.2 Music is a booster in High Tolerant individuals

Our results confirmed the positive impact of music in the High Tolerant group by showing that individuals cycling in music produced greater power output and experienced more positive affective states than those practicing in silence. Furthermore, the dynamic increase in power output across the duration of the session was significantly greater for the group cycling in music than the one cycling in silence. Compared to the few studies in the literature that used a production-mode protocol, our findings are consistent [55]. For example, Elliott et al. [56,57] had participants cycle for 12 minutes on an ergo-cyclometer at an intensity perceived as « somewhat difficult » (RPE 13) in silence, while listening to neutral or to motivational music. Authors observed that the motivational music had the effect to increase the pedalling distance and the positive affective states. In the present case, High Tolerant individuals in the music condition may have used the auditory energizing music to entrain each pedal push to the beat of the music, facilitating the production of the motor task. Furthermore, the importance set on the actual (true) sensory feedback may also have been minimized, i.e. music inhibiting the perception of inner states of discomfort that emerged from the increase in exercise intensity. As a consequence, for similar levels of exercise intensity, the perceived level of discomfort with music was weaker than that experienced when cycling in silence. Exercise intensity could be increased until a match between predicted and true sensory feedback was attained leading High Tolerant individuals to exercise at greater intensities when cycling in music than in silence, at a similar perceived exertion scale of RPE13.

### 4.3 Little effects of music for Low Tolerant individuals

Contrasting results were found in the Low Tolerance groups. Here, whatever the self-reported physical activity level, participants cycling with music produced similar levels of power output and reported similar degrees of positive affective states than those cycling in silence. However, as in the High Tolerant groups, mean heart rate frequency was significantly greater in the group cycling in music than the group cycling in silence. Hence, for the Low Tolerant participants specifically, music induced an increase in heart rate without providing the ability to produce greater power output. In the literature, greater heart rate has been associated to higher perceived exertion [36,58,59]. Hence, the contrasting effects of music on heart rate and power output may be an indicator of the higher difficulties experienced by the Low Tolerant individuals to perform a physical exercise in music rather than in silence. Four contrasting hypotheses may be considered to understand the origin of such difficulty. First, music has an entrainment power that has been well documented in the past ten years [46]. The groove contained within the musical playlists may have increased body rhythmicity [47] and modulated body posture [48]. The entrainment effect of music would have then led Low Tolerant individuals to cycle at a pace too high compared to their predicted acceptable level of discomfort. Another possibility is that participants used cognitive strategies to resist the entrainment effect of the music. Increasing the cognitive load would make the cycling in music task more cognitively challenging than cycling in silence. Individuals would in addition feel a discordance effect, which may increase furthermore the expressed levels of discomfort. Third, music is known to have a distracting effect. By narrowing attention, music can divert the mind from sensations of fatigue and inner senses of discomfort [4,5,60]. In the present case, the dissociation phenomena of music may have turned attention away from detecting the emergence of sensorial negative feelings that cue the forthcoming loss of homeostasis. Finally, set in a high state of arousal, Low Tolerant individuals may have simply been perturbed by the activating effects of music [61]. Further studies are now required to parcel apart the relative contributions of these different possibilities, taking into account tolerance levels to physical effort but also controlling for other factors like the cognitive abilities associated to the planning of sequential motor activities.

### 4.4 Pleasure is an affect that varies over time

Following the writings of Csikszentmihalyi [62], pleasure is an experience that is “homeostatic”, i.e., it incorporates affective states that do not produce psychological growth but satiates biological needs. Pleasurable experiences make us feel good at a given moment. Previous studies have based their assumptions on the fact that changes in affective states, which take place between the start and the end of a session—follow a linear course [19]. However, by assessing the affective states periodically (every 5’), we show here that affective states evolve none linearly through the course of an exercise session. Contrasting affective dynamics may even characterise High and Low Tolerant individuals (see Fig 4) but additional observations are required. More specifically, future studies need to sample affective states throughout the practice sessions at an individual level and with adequate frequency, to gain a better understanding of the possible causal relationship between heart rate frequency, exercise intensity and the observed variations in affective states. As the pleasure experienced in the last 5 minutes of a session is predictive of positive emotional memories [63], the assessment of the changes in affective states during moderate physical activity may be a key variable to target when seeking to convey compliance to regular practice in individuals prone to low thresholds of physical effort and muscle pain.

## 4.5 Concluding remarks

High and Low Tolerant individuals participated in a production-mode protocol in which they were asked to cycle at a moderate intensity felt as « somewhat difficult » (RPE13 on the Borg scale). We show that music is a booster for High Tolerant individuals: the musical environment gave them the ability to produce greater power output while experiencing even more pleasure than when cycling in silence. Low Tolerant individuals experienced with music an increase in heart rate frequency without gain in power output or pleasure, suggesting distress and discomfort when practicing in an energizing environment. Interestingly, music brought greater pleasure to the High than to the Low Tolerant participants even if both groups reported similar levels of enjoyment (PACES at the end of the session). Hence, pleasure and enjoyment may be two different concepts [64] that should be dissociated when seeking to develop pleasurable sports. Additional studies are needed to reveal which of pleasure or enjoyment is the key to promote durable motivation to an active life style in individuals with high and low tolerance to exercise intensity.

## Supporting information

S1 Table Sonic properties of the audio playlists that were used in the cycling in music condition. (DOCX)

S2 Table Demographics. Descriptive results for Age (years), Body Mass Index (kg.m^-2^), Educational Level (number of years after Baccalauréat), Tolerance Score, Repartition of men and women and Repartition of Low and High Tolerant to exercise as a function of experimental conditions.
(DOCX)

S3 Table Self-reported physical activity level. Descriptive results for the quantitative amount of physical activity practiced as a function of experimental group (METs: Metabolic Equivalents—a useful, convenient and standardized way to describe the absolute intensity of a variety of physical activities—ACSM, 2014).
(DOCX)

S4 Table Heart rate frequency statistics. Reports the statistical results for post-hoc analysis conducted on Delta_*Heart Rate Frequency*_ as a function of Assessment period and Experimental Condition (*: p < 0.05, **: p < 0.001).
(DOCX)

S5 Table Power output statistics. Reports the statistical results for post-hoc analysis conducted on Delta_*Power Ouput*_ as a function of Assessment period, Experimental Condition and Tolerance group.
(DOCX)
