# Supplementary material for: Tolerance to exercise intensity modulates pleasure when exercising in music: The upsides of acoustic energy for High Tolerant individuals
Source: PLoS One. 2017 Mar 1;12(3):e0170383. doi: 10.1371/journal.pone.0170383 (PMC5331955; doi:10.1371/journal.pone.0170383)
Supplement: S1 Table — (DOCX) [file pone.0170383.s001.docx]

| KEEP CALM | Tempo | Energy | Groove | Duration | Mode | Valence |
| --- | --- | --- | --- | --- | --- | --- |
| CalmingMusic | 66.014 | 0.125661 | 0.202002 | 299.12644 | 1 | 0.03312 |
| MinimalSteps | 98.031 | 0.088988 | 0.507274 | 330.71002 | 1 | 0.054884 |
| Dreamers | 140.013 | 0.058212 | 0.572493 | 201.86063 | 1 | 0.039156 |
| BlankSpaceFocus | 89.986 | 0.083598 | 0.37934 | 261.55868 | 1 | 0.037322 |
| Balance | 66.751 | 0.105551 | 0.200186 | 282.72729 | 0 | 0.037199 |

| SPORTS | Tempo | Energy | Groove | Duration | Mode | Valence |
| --- | --- | --- | --- | --- | --- | --- |
| Domino | 126.993 | 0.531467 | 0.7753 | 200.82667 | 1 | 0.882473 |
| LoseYourself | 171.387 | 0.762663 | 0.689181 | 326.64 | 1 | 0.060821 |
| Happy | 120.129 | 0.660274 | 0.716196 | 263.49601 | 1 | 0.351701 |
| Stronger | 108.495 | 0.591883 | 0.411862 | 246.10667 | 1 | 0.35792 |
| HeyBrother | 125.012 | 0.790496 | 0.525328 | 206.67955 | 0 | 0.472574 |

| MOTIVATION | Tempo | Energy | Groove | Duration | Mode | Valence |
| --- | --- | --- | --- | --- | --- | --- |
| EyeoftheTiger | 108.72 | 0.377376 | 0.830521 | 250.8 | 0 | 0.619104 |
| LightItUp | 107.985 | 0.87694 | 0.745646 | 166.13823 | 0 | 0.761805 |
| GoodFeeling | 128.006 | 0.853962 | 0.704754 | 247.97333 | 0 | 0.679432 |
| Timber | 130.014 | 0.94247 | 0.586827 | 203.42667 | 1 | 0.799888 |
| YoureOn | 100.1 | 0.866125 | 0.47379 | 171.97288 | 0 | 0.30379 |

| CARDIO | Tempo | Energy | Groove | Duration | Mode | Valence |
| --- | --- | --- | --- | --- | --- | --- |
| LoveMyself | 122.909 | 0.760651 | 0.619438 | 218.77333 | 0 | 0.319986 |
| Beverly | 102.988 | 0.504205 | 0.935589 | 200.38834 | 1 | 0.746023 |
| SunglassesRemix | 117.992 | 0.937084 | 0.738041 | 221.69447 | 1 | 0.843092 |
| Sax | 117.981 | 0.855602 | 0.716741 | 219.54753 | 1 | 0.871007 |
| Backbeat | 139.932 | 0.879355 | 0.707123 | 227.09492 | 0 | 0.681927 |

| WORKOUT | Tempo | Energy | Groove | Duration | Mode | Valence |
| --- | --- | --- | --- | --- | --- | --- |
| CheapThrills | 89.976 | 0.698268 | 0.628313 | 211.66667 | 0 | 0.730264 |
| Sorry | 99.992 | 0.759459 | 0.664727 | 200.78667 | 0 | 0.382132 |
| YOUTH | 91.522 | 0.74946 | 0.626575 | 185.19401 | 1 | 0.565315 |
| CantFeelMyFace | 107.954 | 0.781735 | 0.713659 | 216.46667 | 0 | 0.586261 |
| SomethingSweet | 99.979 | 0.790709 | 0.621856 | 195.56 | 0 | 0.657068 |

S1 Table: **Sonic properties of the audio playlists** that were used in the cycling in music condition
