# Supplementary material for: Tolerance to exercise intensity modulates pleasure when exercising in music: The upsides of acoustic energy for High Tolerant individuals
Source: PLoS One. 2017 Mar 1;12(3):e0170383. doi: 10.1371/journal.pone.0170383 (PMC5331955; doi:10.1371/journal.pone.0170383)
Supplement: S2 Table — Descriptive results for Age (years), Body Mass Index (kg.m-2), Educational Level (number of years after Baccalauréat), Tolerance Score, Repartition of men and women and Repartition of Low and High Tolerant to exercise as a function of experimental conditions. (DOCX) [file pone.0170383.s002.docx]

|  | **Resting Group (N=15)** | | **Cycling Group (N = 24)** | **Cycling in music Group (N = 24)** | **Statistical analysis** |
| --- | --- | --- | --- | --- | --- |
| Age (y) | | 22,8 (6,92) | 24,09 (4,21) | 21,7 (3,43) | F(2,59) = 1.473; p = 0.237 |
| Body mass index (kg.m^-2)^ | | 23,64 (4,35) | 25,38 (5,25) | 25,2 (6,98) | F(2,59) = 0.465, p = 0.630 |
| Educational Level (years) | | 2,6 (1,5) | 3,35 (1,43) | 2,67 (1,73) | F(2,59) = 1.467, p = 0.239 |
| Tolerance Score | | 26,13 (6,78) | 26,75 (4,05) | 26,63 (4,32) | F(2,60) = 0.077, p = 0.926 |
| Repartition of men and women | | Men : N= 4  Women : N= 11 | Men : N= 9  Women : N= 15 | Men : N= 9  Women : N= 15 | F(2,60) = 0.441, p = 0.646 |
| Repartition of Low and High Tolerant to exercise | | Low : N= 10  High : N= 5 | Low : N= 12  High : N= 12 | Low : N= 12  High : N= 12 | F(2,60) = 0.829, p = 0.441. |

S2 Table: Demographics. Reports the descriptive results for Age (years), Body Mass Index (kg.m^-2^), Educational Level (number of years after Baccalauréat), Tolerance Score, Repartition of men and women, and Repartition of Low and High Tolerant to exercise as a function of experimental conditions.
