# Supplementary material for: Tolerance to exercise intensity modulates pleasure when exercising in music: The upsides of acoustic energy for High Tolerant individuals
Source: PLoS One. 2017 Mar 1;12(3):e0170383. doi: 10.1371/journal.pone.0170383 (PMC5331955; doi:10.1371/journal.pone.0170383)
Supplement: S3 Table — Descriptive results for the quantitative amount of physical activity practiced as a function of experimental group (METs: Metabolic Equivalents—a useful, convenient and standardized way to describe the absolute intensity of a variety of physical activities—ACSM, 2014). (DOCX) [file pone.0170383.s003.docx]

|  | **Resting Group (N=15)** | **Cycling in silence (N = 24)** | **Cycling in music (N = 24)** | **Statistical analysis** |
| --- | --- | --- | --- | --- |
| Total Low Physical Activity (METs-minutes/week) | 1087 (812) | 757 (688) | 905 (1014) | F(2,61) = 0.686; p = 0.507 |
| Total Moderate Physical Activity (METs-minutes/week) | 457 (340) | 617 (734) | 751 (1079) | F(2,61) = 0.594; p = 0.555 |
| Total Vigorous Physical Activity (METs-minutes/week) | 944 (1366) | 1145 (1391) | 1641 (2224) | F(2,61) = 0.897 p = 0.425 |
| Total Physical Activity Practiced (METs-minutes/week) | 2488 (1594) | 2520 (1813) | 3298 (3789) | F(2,61) = 0.637; p = 0.532 |

S3 Table: Fitness level. Descriptive results for the quantitative amount of physical activity practiced as a function of experimental group (METs: Metabolic Equivalents - a useful, convenient and standardized way to describe the absolute intensity of a variety of physical activities - ACSM, 2014).
