# Supplementary material for: Tolerance to exercise intensity modulates pleasure when exercising in music: The upsides of acoustic energy for High Tolerant individuals
Source: PLoS One. 2017 Mar 1;12(3):e0170383. doi: 10.1371/journal.pone.0170383 (PMC5331955; doi:10.1371/journal.pone.0170383)
Supplement: S4 Table — Reports the statistical results for post-hoc analysis conducted on DeltaHeart Rate Frequency as a function of Assessment period and Experimental Condition (*: p < 0.05, **: p < 0.001). (DOCX) [file pone.0170383.s004.docx]

|  | **At 5 min** | **At 10 min** | | **At 15 min** | | **At 20 min** | **At 25 min** |
| --- | --- | --- | --- | --- | --- | --- | --- |
| *Cycling in silence group*  10’  15’  20’  25’  30’ | -5.58 ± 2.67*  -13.13 ± 3.49*  -14.72 ± 4.21*  -19.59 ± 4.39*  -14.63 ± 4.72 | | -4.55 ±1.65  -6.14 ± 2.42  -11 ± 2.74*  -6.05 ± 3.36 | | -1.59 ± 1.7  -6.46 ± 2.06  -1.5 ± 2.83 | -4.87 ± 1.29*  0.87 ± 2.48 | 4.96 ± 2.37 |
| *Cycling in music group*  10’  15’  20’  25’  30’ | -15.81 ± 2.52**  -22.24 ± 3.31**  -27.25 ± 3.98**  -30.17 ± 4.15**  -32.4 ± 4.46** | | -6.43 ± 1.57*  -11.44 ± 2.29**  -14.36 ± 2.6**  -16.59 ± 3.18** | | -5.02 ± 1.61  -7.93 ± 1.95*  -10.16 ± 2.69* | -2.91 ± 1.22  -5.15 ± 2.35 | 2.23 ± 2.24 |

S4 Table: Heart Rate frequency statistics. Reports the statistical results for post-hoc analysis conducted on Delta*_Heart Rate Frequency_* as a function of Assessment period and Experimental Condition (* : p < 0.05, **: p < 0.001).
