# Supplementary material for: Tolerance to exercise intensity modulates pleasure when exercising in music: The upsides of acoustic energy for High Tolerant individuals
Source: PLoS One. 2017 Mar 1;12(3):e0170383. doi: 10.1371/journal.pone.0170383 (PMC5331955; doi:10.1371/journal.pone.0170383)
Supplement: S5 Table — Reports the statistical results for post-hoc analysis conducted on DeltaPower Ouput as a function of Assessment period, Experimental Condition and Tolerance group. (DOCX) [file pone.0170383.s005.docx]

|  | 5’ | 10’ | 15’ | 20’ | 25’ |
| --- | --- | --- | --- | --- | --- |
| ***Cycling in silence group***  *High Tolerance group*  10’  15’  20’  25’  30’  *Low Tolerance group*  10’  15’  20’  25’  30’ | -4.42 ± 2.27  -5.5 ± 2.97  -4.58 ± 3.53  -0.92 ± 4.31  -5.83 ± 6.05  1.5 ± 2.28  -2.17 ± 2.97  -1.25 ± 3.53  -6.83 ± 4.31  -0.92 ± 6.05 | -1.08 ± 1.68  -0.17 ± 2.42  3.5 ± 3.66  -1.42 ± 5.49  -3.67 ±1.68  -2.75 ± 2.42  -8.33 ± 3.66  -2.42 ± 5.49 | 0.97 ± 2  4.59 ± 2.96  -0.33 ± 5.48  0.97 ± 2  -4.67 ± 2.96  -1.25 ± 5.48 | 3.67 ± 2.55  -1.25 ± 5.45  -5.58 ± 2.55  0.33± 5.45 | 4.92 ± 5.19  5.92 ± 5.19 |
| ***Cycling in music group***  *High Tolerance group*  10’  15’  20’  25’  30’  *Low Tolerance group*  10’  15’  20’  25’  30’ | -2.83 ± 2.27  - 4.83 ± 2.97  - 8.25 ± 3.53  - 11.42 ± 4.31  - 22.92 ± 6.05*  -3.25 ± 2.27  - 4 ± 2.97  -2.33 ± 3.53  -2.83 ± 4.31  7.08 ± 6.05 | - 2 ± 1.68  - 5.42 ± 2.42  - 8.58 ± 3.66  -20.08 ± 5.49*  3.25 ± 2.28  0.75 ± 1.68  -0.92 ± 2.42  10.3 ± 5.49 | - 3.42 ± 2  - 6.58. ± 2.96  - 18.08 ± 5.48*  1.67 ± 2  - 1.17 ± 2.96  11.08 ± 5.48 | -3.17 ± 2.55  -14.67 ± 5.45  -0.5 ± 2.55  9.42 ± 5.45 | -11.5 ± 5.19  9.92 ± 5.19 |

S5 Table: Power Output statistics. Reports the statistical results for post-hoc analysis conducted on Delta*_Power Ouput_* as a function of Assessment period, Experimental Condition and Tolerance group.
